# The Application of Newcastle Disease Virus (NDV): Vaccine Vectors and Tumor Therapy

**DOI:** 10.3390/v16060886

**Published:** 2024-05-30

**Authors:** Huiming Yang, Jiaxin Tian, Jing Zhao, Ye Zhao, Guozhong Zhang

**Affiliations:** 1 National Key Laboratory of Veterinary Public Health Security, College of Veterinary Medicine, China Agricultural University, Beijing 100193, China; yanghuiming12@126.com (H.Y.); tianjiaxin323@163.com (J.T.); zhaoj@cau.edu.cn (J.Z.); yezhao@cau.edu.cn (Y.Z.); 2 Key Laboratory of Animal Epidemiology of the Ministry of Agriculture, College of Veterinary Medicine, China Agricultural University, Beijing 100193, China

**Keywords:** Newcastle disease virus, vaccine vector, tumor therapy

## Abstract

Newcastle disease virus (NDV) is an avian pathogen with an unsegmented negative-strand RNA genome that belongs to the Paramyxoviridae family. While primarily pathogenic in birds, NDV presents no threat to human health, rendering it a safe candidate for various biomedical applications. Extensive research has highlighted the potential of NDV as a vector for vaccine development and gene therapy, owing to its transcriptional modularity, low recombination rate, and lack of a DNA phase during replication. Furthermore, NDV exhibits oncolytic capabilities, efficiently eliciting antitumor immune responses, thereby positioning it as a promising therapeutic agent for cancer treatment. This article comprehensively reviews the biological characteristics of NDV, elucidates the molecular mechanisms underlying its oncolytic properties, and discusses its applications in the fields of vaccine vector development and tumor therapy.

## 1. Introduction

Newcastle disease (ND) is one of the highly pathogenic viral diseases of avian species. The causative agent, Newcastle disease virus (NDV), is an avian paramyxovirus capable of causing serve respiratory, gastrointestinal, and neurological disorders in poultry, leading to enormous economic losses for the poultry industry worldwide [1]. As a cytoplasmic virus that does not integrate into the host genome or undergo recombination, NDV exhibits a remarkable tropism for neoplastic cells, inducing their lysis while sparing normal cells [2]. This tumor-selective replication, coupled with the absence of pre-existing immunity in humans due to its host range restriction, renders NDV an attractive candidate for oncolytic virotherapy. Beyond directly lysing tumor cells, NDV can engage various signaling pathways to induce autophagy, inflammation, necrosis, ferroptosis, apoptosis, and immunogenic cell death [3,4]. Additionally, it can stimulate both innate and adaptive antitumor immune responses, enhancing its oncolytic effects [5,6]. Since the first virus strain was obtained through reverse genetics in 1999 [7], and the first generation of recombinant NDV expressing a foreign gene in 2000 [8], the genetic manipulations of various viral strains have made significant progress. To date, numerous NDV-vectored vaccines expressing protective antigens from various pathogens have been generated, and the virus has also been genetically reprogrammed to improve its oncolytic efficacy against a variety of human cancers.

This review elaborates on the biological characteristics of NDV, the molecular mechanism underlying its oncolytic properties, and its application in vaccine vector development and tumor therapy.

## 2. Molecular Biology of NDV

NDV, also known as avian paramyxovirus 1 (APMV-1), is a single-stranded, negative-sense RNA [ssRNA(-)] virus with a lipid bilayer. According to the updated unified phylogenetic classification system and revised nomenclature for NDV, it was categorized as avian orthoavulavirus 1 of the *Orthoavulavirus* genus, Avulavirinae subfamily, and Paramyxoviridae family. The genome is composed of six transcriptional units that encode six main viral proteins: nucleocapsid protein (NP), phosphoprotein (P), matrix protein (M), fusion protein (F), hemagglutinin-neuraminidase protein (HN), and large polymerase protein (L). As a result of RNA editing of the P gene, two accessory proteins V and W are produced [9]. The V protein can antagonize the interferon (IFN) pathway in avian hosts but not in mammalian cells [10,11,12]. The W protein can be expressed in the nucleus or the cytoplasm depending on the genotype of the viral strain [13]. Among its structural proteins, the NP, P, and L proteins form the ribonucleoprotein complex (RNP) that embeds the genomic RNA, which is responsible for the replication of the virus. M protein forms the inner layer of the envelope and is involved in viral assembly and budding. The HN and F proteins are surface glycoproteins in the form of oligomers, which together with the lipid bilayer membrane of the host constitute the outer envelope of the virus and participate in the entry of the virus into cells.

**Figure 1:**
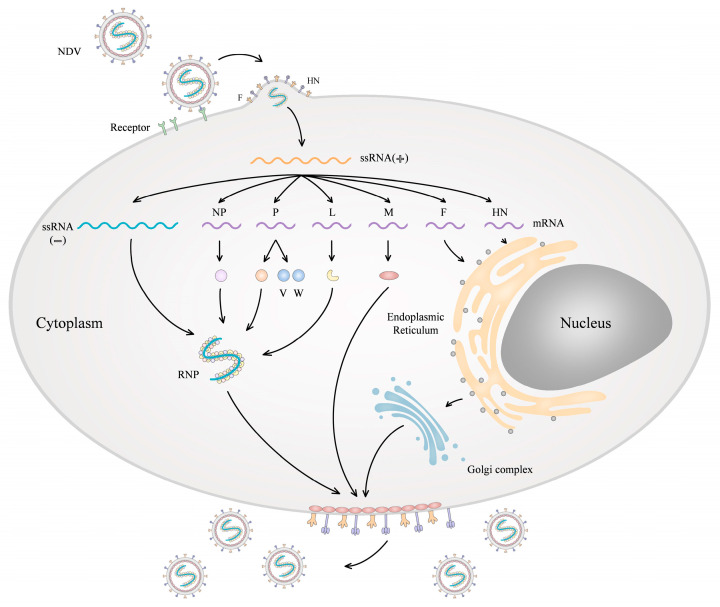
The process of cell infection by NDV. The NDV hemagglutinin-neuraminidase (HN) protein binds to sialic acid receptors on the host cell surface, facilitating membrane fusion mediated by the fusion (F) protein. This allows entry of the viral ribonucleoprotein (RNP) complex into the cytoplasm. The viral RNA-dependent RNA polymerase transcribes the negative-sense single-stranded viral genomic RNA into positive-sense mRNA, which serves as a template for the translation of viral proteins. Viral genome replication also occurs in the cytoplasm. Newly synthesized genomic RNA associates with nucleoprotein (NP), phosphoprotein (P), and large polymerase (L) proteins to form neo-RNP complexes. These complexes are assembled with the F, HN, and matrix (M) proteins at the host cell plasma membrane, leading to the budding and release of progeny virions.

The main process by which NDV infects host cells is as follows (Figure 1). HN protein can trigger the activation and conformational change of the F protein. This occurs through binding to sialic acid receptors on the surface of the host cells to promote fusion of the viral envelope and cell plasma membrane and allow the RNP to enter the cytoplasm of a host cell [1]. NDV can also enter cells via clathrin-mediated endocytosis [14], micropinocytosis [14], and RhoA-dependent endocytosis [15]. The viral genome replicates in the cytoplasm. The genomic ssRNA(-) is transcribed into mRNAs that are then translated into different viral proteins. The full-length, anti-genomic RNA (positive-strand) is then used as a template for the synthesis of genomic ssRNA(-). The newly formed genomic RNA is then enveloped in NP, P, and L proteins to form the RNP which is assembled with matrix and surface glycoproteins and then released from the host cell [1]. Finally, HN protein can remove sialic acid residues from the nascent virions, preventing their aggregation and promoting viral spread within the infected tissues [16].

NDV mainly infects birds and does not infect mammals, including humans, although it may cause minor transient symptoms in humans, such as conjunctivitis and flu-like symptoms. According to their pathogenicity and virulence in chickens, NDV strains can be divided into three types: lentogenic (no disease), mesogenic (moderate-to-severe disease), and velogenic (severe disease with high mortality). In the most recent phylogenetic classification system, NDVs are subdivided into two categories, namely class I and class II. Class I contains one unique genotype (genotype 1), while class II contains 21 genotypes (I–XXI) [17]. However, different strains are not distinguishable by serology. In general, the cleavage site of F protein mainly determines the virulence of NDV [18,19]. The F cleavage site of velogenic and mesogenic strains usually has a polybasic amino acid structure, which is recognized and cleaved by Folin-like proteases, exhibiting superior capacity for multicyclic replication, syncytium formation, and tumor cell lysis, inducing deadly respiratory and gastrointestinal diseases in birds. Lentogenic strains have a single amino acid motif, which is cleaved by extracellular trypsin-like proteases, exhibiting reduced capacity for multicyclic replication and lysis [20,21].

## 3. Advantages of NDV as a Vaccine Carrier

NDV is an attractive vaccine vector candidate for both human and animal use, especially lentogenic strains of NDV. The following properties of NDV can be attributed to its credibility as a viral vector: (1) NDV grows to high titer in chicken embryos and cell cultures, which is convenient for large-scale production. (2) NDV has a modular genome, with only six essential genes, that is easy to manipulate and can stably accommodate and express foreign genes. (3) The risk of gene exchange and recombination is low. NDV replicates in the cytoplasm and the viral genome does not integrate with the host genome in the nucleus. (4) NDV can elicit a systematic immune response, including mucosal, humoral, and cellular immunity. (5) NDV is highly host-restricted and infects birds naturally. There is no NDV-specific pre-existing immunity in mammals, including humans, which is an advantage of NDV-vectored vaccines in these hosts.

## 4. Advantages of NDV as an Oncolytic Agent

NDV has oncolytic properties and can effectively stimulate an antitumor immune response, which makes it a promising antitumor treatment candidate. Compared with other oncolytic viruses, NDV has the following characteristics: (1) NDV comprises a single negative strand that replicates in the cytoplasm, and there is no DNA stage in the replication process. Cytoplasmic replication means that the virus is independent of the host cell DNA replication mechanism, is unable to integrate with the host genome, and does not recombine with human viruses. (2) NDV has low production costs, can be administered via diverse routes, and has few side effects. (3) NDV can be selected to replicate efficiently in tumors and does not replicate efficiently in normal cells of non-avian hosts. NDV replication in tumor stem cells and dormant tumor cells may not be affected by radiotherapy or chemotherapy because NDV replication is independent of cell proliferation [22]. (4) In addition to playing a direct oncolytic role, NDV can also promote the activation of the immune system and exert antitumor activity.

## 5. Oncolytic Mechanism of NDV

**Figure 2:**
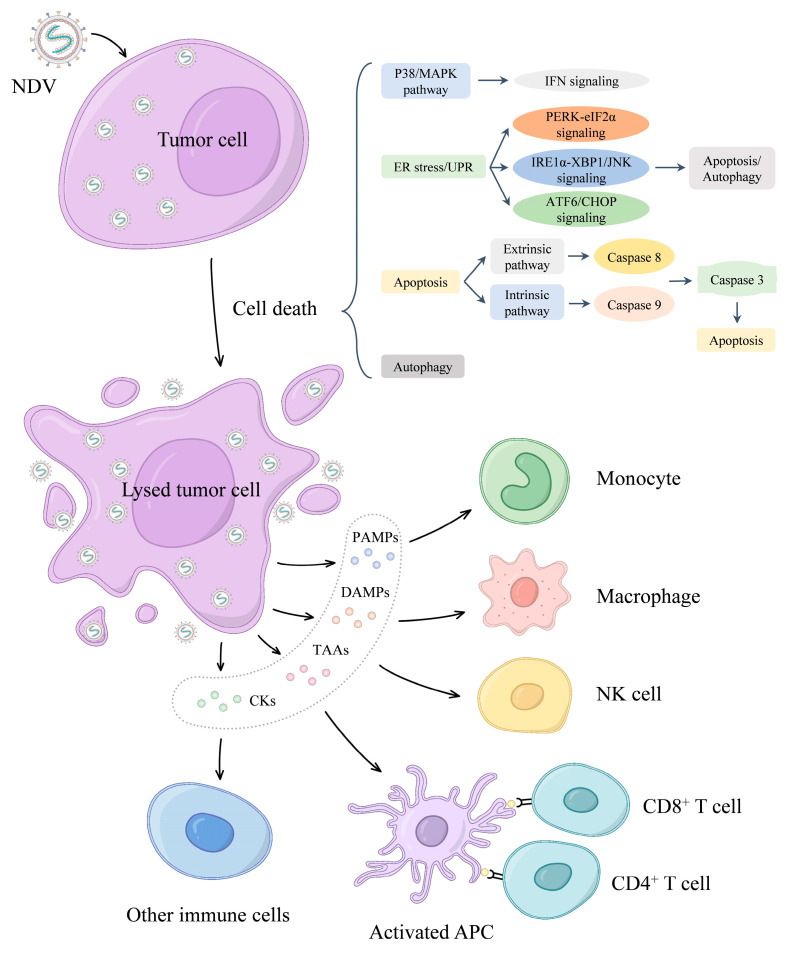
Tumor cell death and the antitumor immune response induced by NDV. Upon infecting tumor cells, NDV mainly regulates cell death through the P38/MAPK pathway, endoplasmic reticulum (ER) stress, apoptosis, and autophagy. Consequently, the release of pathogen-associated molecular patterns (PAMPs), damage-associated molecular patterns (DAMPs), tumor-associated antigens (TAAs), and cytokines (CKs) from lysed tumor cells activates innate immune cells such as natural killer (NK) cells, monocytes, and macrophages. Moreover, these molecules stimulate the activation of tumor-specific CD4^+^ and CD8^+^ T cells and recruit antigen-presenting cells (APCs) into the tumor microenvironment, thereby initiating an antitumor immune response.

Since Cassel et al. first reported the oncolytic effects of NDV in 1965 [23], NDV has garnered significant attention as an oncolytic agent. Numerous preclinical and clinical investigations have validated and examined NDV in diverse cancer models in animals and humans [22,24]. NDV selectively infects tumor cells and induces tumor cell death (oncolysis). Initially, NDV elicits direct cytolytic effects on infected tumor cells. Subsequently, immunogenic cell death pathways activated by viral oncolysis stimulate systemic antitumor immune responses (Figure 2). Emerging evidence indicates that NDV has the ability to disrupt cancer cell metabolism [25,26].

### 5.1. Tumor-Selective Viral Replication

As mentioned above, NDV infects cells via two steps: (1) cell binding, membrane fusion, viral genome transduction, and viral gene transcription; and (2) viral replication using a plus-strand full-length template [1]. The first step occurs in a wide range of cell types, whereas the second step only occurs in the tumor cells of non-avian hosts, such as mice or humans, because viral replication is inhibited in normal cells by defense mechanisms involving type I IFN (IFN-I). It was reported that NDV replicates 10,000 times faster in human tumor cells than in most normal human cells [27]. The main reasons for this difference are defects in the IFN-I signaling pathways and insensitivity to the IFN-I receptor-mediated signaling pathway in tumor cells [28,29,30]. The key components of the IFN-I signaling pathway in tumor cells, such as cytoplasmic protein kinase dsRNA activation (PKR), retinoic acid inducible gene I (RIG-I), interferon regulatory factors (IRFs), and cell surface IFN-I α receptors (IFNAR), may be downregulated, which prevents the normal IFN response after NDV infection, leading to viral replication and spread within cells [2,31,32,33]. In addition, tumor-selective replication of NDV was found to be associated with antiviral response defects. Fiola et al. analyzed tumor cells infected with NDV and found several defects in antiviral response pathways, including delayed activation of antiviral proteins and a lack of response to UV-inactivated NDV [30]. The expression levels of antiviral genes (RIG-1, IRF-3, IRF-7, and IFN-B) are associated with susceptibility to NDV [34]. NDV is more likely to infect cells with high levels of antiviral gene expression. When the expression level of antiviral genes is low, the infection rate of NDV is also low. Tumor cell susceptibility to NDV may also be based on overexpression of tumor cell surface molecules, such as cell surface proteins containing sialic acid [35,36]. Defects in the apoptotic pathway in tumor cells also contribute to the specific targeting of NDV to tumor cells. Overexpression of anti-apoptotic proteins BcL-xL and Livin increases the susceptibility of tumor cells to NDV, while viral replication increases significantly [37,38]. A recent study showed that the small Rho GTPase Rac1 is targeted by NDV in human transformed tumorigenic cell lines [39]. Rho GTPases belong to the branch of small GTPases of the Ras superfamily of oncogenes. Rac1 gene downregulation leads to the inhibition of NDV replication [40]. Overactive Ras blocks PKR, which promotes the tumor-selective replication of oncolytic viruses [41].

### 5.2. NDV Mediates Oncolysis

#### 5.2.1. Apoptosis

Apoptosis is a highly regulated form of programmed cell death and one of the most important cellular defense mechanisms of host cells against viral infection [42]. As members of the cysteine protease family, caspases are central regulatory factors that play an important role in the process of apoptosis, and caspase 3, caspase 8, and caspase 9 are the most important apoptotic signal transduction proteins [43]. NDV infection induces the apoptosis of tumor cells mainly through extrinsic and intrinsic pathways. The mitochondrial pathway (intrinsic pathway) activates caspase 9, and the extrinsic pathway activates caspase 8 [43]. Loss of mitochondrial membrane potential, release of cytochrome C, and activation of caspase 9 are the basic elements of the mitochondrial apoptotic pathway. This pathway is mainly regulated by the Bcl-2 protein family, which includes anti-apoptotic members and pro-apoptotic members [44]. The ratio of Bax/Bcl-2 factors can identify the level of NDV-induced apoptosis [45,46]. The exogenous apoptotic pathway is activated by binding of cytokine ligands (FasL, TNF-α, and TRAIL) to corresponding tumor necrosis factor receptors (TNFRs) on the cell surface. The overexpression of Fas and TRAIL can increase the oncolytic effect of NDV [47,48]. The synergistic effects of TRAIL secreted by drug administration and the NDV strain MTH-68/H also promote the death of tumor cells [49]. In addition, the MAPK and endoplasmic reticulum (ER) stress pathways play important roles in NDV-mediated tumor lysis. NDV strain AF2240 induces apoptosis through the P38 MAPK/NF-κB/IκBα pathway in renal carcinoma cells [50]. The ER stress response, also known as the unfolded protein response (UPR), leads to the aggregation of new peptides or unfolded proteins in the ER, which in turn activates a series of signaling pathways, such as PERK-eIF2α, ATF6, and IRE1α, and may ultimately induce the expression of pro-apoptotic protein CHOP, thereby inducing apoptosis. It was reported that eIF2α-CHOP-BcL-2/JNK and IRE1α-XBP1/JNK signaling can promote apoptosis and inflammation and support the proliferation of NDV [51].

Cell lines that respond to endogenous or exogenous IFN and those with an impaired IFN response undergo apoptosis after NDV infection, suggesting that NDV-induced apoptosis is independent of IFN signaling [52]. Specific blocking of the receptor-mediated endocytosis pathway, UV inactivation of viral replication, or blocking of the viral translation process all effectively reduce NDV-induced apoptosis, suggesting that NDV-induced apoptosis requires viral replication and protein expression [1,3,31]. The envelope protein of NDV is mainly involved in the apoptotic process. HN protein can induce human peripheral blood mononuclear cells (PBMCs) to upregulate the expression of tumor necrosis factor-associated apoptosis ligand (TRAIL) [53,54]. HN gene expression alone has been reported to induce apoptosis in human breast cancer MCF-7 cells [55]. A novel oncolytic adenovirus (Ad-hTERTp-E1a-HN) expressing NDV-HN protein can selectively inhibit esophageal cancer EC-109 cells and inhibit tumor growth in mice [56]. The M protein has been reported to interact with Bax through its BH3 domain, resulting in activation of the endogenous apoptotic pathways [46]. BH-like domains were also found in F, L, and HN proteins, but only the overexpression of NDV F protein in HeLa cells led to an increase in cell death [57].

Some apoptosis-related proteins, antiviral proteins, and immune-related proteins also affect NDV-mediated apoptosis. For example, estrogen receptor α (ERα) modulates apoptosis in breast cancer cells in response to estrogen. NDV strain D90 could further promote estrogen-mediated apoptosis in ERα-positive cells [58]. Recombinant NDV expressing p53 induced the apoptosis of glioma cells through upregulation of apoptosis-related genes [59]. The antiviral protein ISG12(1) could induce apoptosis by redistributing Bax to inhibit NDV replication [60]. The TXNL1 protein induced apoptosis in DF-1 cells via a pathway involving Bcl-2/Bax and caspase 3 [61].

#### 5.2.2. Autophagy

Autophagy is a cellular process involving the formation of double-membrane vesicles that transport intracellular material to lysosomes for degradation and recycling. Various stress factors, such as nutrient deficiency, endoplasmic reticulum stress, oxidative stress, and viral infection, regulate the occurrence of autophagy. At the molecular level, a series of autophagy-related genes (ATGs) carefully regulate the initiation, nucleation, elongation, and maturation of autophagy. The mammalian target of rapamycin (mTOR) and AMP-activated protein kinase (AMPK) are two key signaling pathways involved in the regulation of autophagy [62]. Under adequate nutrition, mTOR inhibits autophagy by suppressing the autophagy initiation kinase ULK1 complex. Under stress conditions such as starvation, AMPK is activated and inhibits mTOR activity, thereby releasing the inhibition of the ULK1 complex and initiating autophagy.

As a crucial component of the host defense system, autophagy plays a dual role in tumor development. Autophagy can maintain cellular homeostasis by removing harmful substances such as damaged organelles and misfolded proteins, thus inhibiting tumor formation. Conversely, autophagy can provide nutrients and energy for tumor cells, helping them adapt to adverse environments such as hypoxia and nutrient deprivation, thereby promoting tumor progression. Furthermore, autophagy induced by viral infection can activate antitumor immunity by enhancing the processing and presentation of tumor antigens by dendritic cells. Given the role of autophagy in tumor progression, autophagy inhibitors such as chloroquine and its derivative hydroxychloroquine have been used in antitumor therapy [63].

NDV infection has been found to induce autophagy in various cell types, such as human glioma cells [64,65] and chicken embryo fibroblasts [66], with increased levels of autophagy favoring viral replication in host cells. Studies have shown that the NP and P proteins of NDV could induce autophagy through the ER stress-related UPR response [67]. F and HN proteins are also involved in autophagy via activation of the AMPK-mTORC1-ULK1 pathway [68]. Additionally, NDV infection can trigger autophagy by regulating multiple UPR signaling pathways, such as PERK/eIF2α, IRE1/JNK, and ATF6/CHOP [69,70,71,72].

Autophagy plays a complex and important role in NDV replication. It can directly promote NDV replication in host cells and provide a more favorable environment for viral replication by regulating host cell metabolism. In NDV-infected glioma cells, inhibition of autophagy through treatment with the autophagy inhibitor chloroquine or siRNA-mediated silencing of autophagy-associated genes BECN1 and ATG5 significantly reduced viral replication. Conversely, treating cells with the autophagy inducer rapamycin enhanced NDV spread [64]. In addition to directly affecting viral replication, autophagy can promote NDV proliferation by reshaping host cell metabolism [73]. Studies have found that NDV infection can induce mitophagy, transporting damaged mitochondria to lysosomes for degradation, thereby reducing cytochrome C release and inhibiting caspase-dependent apoptosis [74]. Kang et al. demonstrated that apoptosis inhibition enhanced autophagy and promoted cell survival and NDV replication [75]. Beclin-1, a core molecule responsible for regulating autophagosomes in post-infection autophagy, has a BH3 binding domain and can bind to a variety of anti-apoptotic Bcl-2 family proteins, which may have direct pro-apoptotic and anti-autophagic effects on tumor cells [76]. When the level of autophagy induced by drug action on tumor cells is increased, the Beclin-1-Bcl-2 complex may be dissociated, thus producing autophagy-promoting and anti-apoptotic effects on tumor cells [76]. This may be one of the molecular mechanisms underlying the combined use of NDV and autophagy inducers in tumor treatment.

#### 5.2.3. NDV Activates the Antitumor Immune Response

After selectively infecting tumor cells, NDV can not only directly kill the tumor, but also stimulate the body’s immune response and enhance the antitumor effect. On the one hand, NDV infection directly activates non-specific immune cells such as natural killer cells [77], monocytes [78], macrophages [79], and dendritic cells [80]. These cytolytic and phagocytic immune cells target infected tumor cells resisting viral lysis. On the other hand, NDV infection can activate the type I IFN signaling pathway, and although tumor cells usually exhibit impaired type I IFN signaling, the damage caused by type I interferons is usually not absolute. Additionally, since NDV can infect normal cells in the tumor microenvironment, a type I IFN response can be elicited even after infection. Transcriptomic analysis of mouse tumors after injection of NDV showed that upregulation of type I IFN response-related genes and a series of cytokines and chemokines mediated the recruitment and proliferation of innate and adaptive immune cells, contributing to antitumor immunity [81]. Furthermore, NDV induces immunologic cell death (ICD), a concept in tumor cell death that involves the activation of the immune system against cancer in immunocompetent hosts. Upon infection with oncolytic viruses, tumor cells release pathogen-associated molecular patterns (PAMPs), damage-associated molecular patterns (DAMPs), tumor-associated antigens (TAAs), cytokines (CKs), and other immunogenic molecules. These factors can not only activate innate immune cells, but also activate tumor-specific T cells, and recruit antigen-presenting cells into the tumor to initiate an immune response [2]. Dendritic cells and antigen-specific CD8^+^ T cells are key effector cells that initiate antitumor effects [82,83]. Moreover, inflammation ensuing from NDV infection assists immune-mediated tumor clearance. However, the concomitant antiviral immune response may also limit oncolytic viral activity [84]. Elucidating the delicate balance between antiviral and antitumor immunity will inform strategies to maximize the immunotherapeutic potential of NDV in cancer treatment.

## 6. The Application of NDV in Vaccine Vector and Tumor Therapy

### 6.1. Application of NDV as a Vaccine Carrier in Infectious Diseases

NDV has been explored as a vector for veterinary and human vaccines, with over 100 published studies on vaccine candidates over the past two decades. Other reviews have listed these studies previously [85,86,87,88]. Traditional low-virulence NDV strains or modified live vaccines dominated by low-virulence NDV strains are usually used as vaccine carriers. The following are examples of animal and human diseases for which vaccine antigens have been successfully delivered using NDV.

For poultry, NDV was used as a carrier in the form of a bivalent vaccine. Namely, NDV expressed antigens of another avian virus to immunize chickens against both diseases. The avian viruses included the highly pathogenic influenza virus [86], avian reovirus [89], infectious bronchitis virus [90,91], infectious laryngotracheitis virus [92], infectious bursal disease virus [93,94], and fowl adenovirus serotype 4 [95]. In addition, recombinant NDV vaccines against duck Tembusu virus [96], goose parvovirus [97], and goose astrovirus [98] have also been developed. Recently, NDV was used as a vector for the production of new vaccines for pigs, cattle, cats, and dogs. The recombinant NDVs expressing the E2 and ERNS genes of classical swine fever virus [99], glycoprotein 3 and/or 5 genes of porcine reproductive and respiratory syndrome virus [100], glycoprotein gene of bovine ephemeral fever virus [101], H protein of canine distemper virus [102], and glycoprotein of rabies virus [103] showed good immunogenicity in animals. In the field of human medicine, NDV-vectored vaccine candidates have been used to develop vaccines against a variety of pathogens, including HIV, EBOV, poliovirus, Japanese encephalitis, influenza, and SARS-CoV-2 [87]. Effective protection against the corresponding pathogen has been shown in animals, indicating that NDV-vectored vaccines have great potential in preventing these diseases [104,105,106,107,108,109]. These findings suggest that NDV is a promising vector for the development of new human vaccines. However, NDV vectors for human disease have mostly been evaluated in animal models, and human trials are needed to assess the safety, immunogenicity, and effectiveness of such vaccines. Due to the COVID-19 pandemic, several clinical trials of NDV-vectored vaccines against SARS-CoV-2 have been conducted. These include the Patria live vaccine in Mexico, inactivated HXP-GPOVac vaccine in Thailand, inactivated COVIVAC vaccine in Vietnam, and inactivated ButanVac vaccine in Brazil. These vaccines were developed by expressing the HXP-S antigen of SARS-CoV-2 in the La Sota strain and have demonstrated some protective effects [87].

### 6.2. Application of NDV in Tumor Therapy

Since the oncolytic properties of NDV were discovered in the 1950s, it has been widely used in preclinical research as a novel anticancer drug for a variety of solid tumors and resistant tumors, such as gastric cancer [110,111], liver cancer [112], lung cancer [113], breast cancer [58], cervical cancer [114], prostate cancer [115], colorectal cancer [116], and glioblastoma [117]. To date, we found 15 clinical trials where NDV was used for cancer therapy (Table 1). Interestingly, most of the clinical trials with NDV were completed in the late 1990s, with only two registered. Compared with other oncolytic viruses, there is still a long way to go before NDV viral therapy is approved and entered into the market [118].

The major NDV strains evaluated for direct human injection were 73-T [23], MTH-68/H [119], PV-701 [120], HUJ [121], and ATV-NDV [122]. The first report of using NDV to treat a human cancer (acute leukemia) was published in 1964, and the patient experienced a brief anti-leukemic effect and clinical symptom remission [123]. In 1965, Cassel et al. used the NDV 73-T strain as an antitumor agent to treat cervical cancer, and the results showed extensive tumor shedding and reduced supraclavicular lymph node metastasis [23]. Subsequently, they employed NDV as an adjuvant immunotherapy in the postoperative treatment of stage II and III malignant melanoma, and the recent long-term follow-up of 83 patients receiving treatment showed that the 10-year survival rate was more than 60% and the 15-year survival rate was 55% [124,125]. In 1993, Csatary conducted a placebo-controlled phase II clinical trial using the NDV MTH-68/H strain via inhalation, which demonstrated a two-year survival rate of 21% in the NDV-treated group and 0% in the placebo group [126]. Another study reported that four patients with high-grade glioblastoma treated with MTH-68/H experienced disease remission and improved survival [127]. The NDV PV701 and HUJ strains also shown good efficacy in clinical applications [120,121,128,129]. The Malaysian NDV strain AF2240, initially developed as a vaccine, has emerged as a promising oncolytic agent for research [130]. Studies have demonstrated the enhanced sensitivity of MDA-MB-231 breast cancer cells to AF2240-mediated oncolysis compared to MCF-7 cells. Infection of cancer cells with this strain induces characteristic apoptotic morphological changes, including cellular atrophy, nuclear fragmentation, chromatin condensation, membrane blebbing, and the formation of apoptotic bodies. Notably, the AF2240 strain exhibits potent oncolytic activity even under hypoxic conditions, a hallmark of the tumor microenvironment, further underscoring its therapeutic potential [55,131,132].

With the concept of a “live cell vaccine”, an autologous tumor cell vaccine modified by infection with NDV (ATV-NDV) was developed. A clinical study [133] evaluated the effects of the vaccine’s quality parameters on the survival of early breast cancer patients treated postoperatively with ATV-NDV. The results showed that the overall survival four years after surgery was 96% for patients who received a high-quality vaccine (*n* = 32), compared to an overall survival of 68% for those who had received a low-quality vaccine (*n* = 31). Other clinical trials of ATV-NDV have shown promising results for the treatment of locally advanced renal cell carcinoma [134], head and neck squamous cell carcinoma (HNSCC) [135], glioblastoma multiforme (GBM) [136], and resected colorectal carcinoma [137,138,139,140].

With the advent of reverse genetics, strategies for improving the oncolytic efficacy of the virus have emerged. One such strategy is reprogramming the virus to express interferons, pro-inflammatory cytokines, or antitumor factors. Numpadit et al. constructed a recombinant NDV (rNDV-IFNγ) that would release IFN-γ and target melanoma cells, showing a stronger oncolytic effect [141]. Additionally, a recombinant NDV expressing IL-2 and IL-12 has been shown to enhance antihepatoma activity in mice [142], and a recombinant NDV co-expressing IL-7 and IL-15 exhibited effective, potent, antitumor properties against melanoma cells in mice [143]. Furthermore, recombinant NDV expressing IL-24 showed high oncolytic efficacy in mouse melanoma models [144]. In another study, the recombinant NDV strain AF2240 engineered to express IL-12 was found to exert strong cytotoxic effects in combating colon cancer [131,132]. Moreover, NDV was engineered to express MIP3α or mOX40L, and both recombinant viruses had higher antitumor activity than the wild-type virus [145,146]. Furthermore, the combination of NDV and various cancer drugs can fully activate the innate and adaptive antitumor immunity of the body. Immune checkpoint inhibitors are one of the most promising classes of drugs in tumor therapy in recent years [147]. Immune checkpoint inhibitors include programmed cell death protein 1 (PD-1) and cytotoxic T-lymphocyte associated protein 4 (CTLA-4). It was reported that recombinant NDV expressing CTLA-4 worked together with radiotherapy to enhance tumor clearance of murine melanoma [148]. Durvalumab is a selective, high-affinity, human IgG1, monoclonal antibody that blocks programmed death-ligand 1 (PD-L1) binding to PD-1 [149]. At present, a recombinant NDV (73-T strain) expressing granulocyte-macrophage colony-stimulating factor (GM-CSF) (MEDI5395) has been used in combination with durvalumab to treat patients with various advanced malignancies. The clinical results (NCT03889275) showed that the secretion of pro-inflammatory cytokines, such as IL-6, IL-8 and IFN-α, in human PBMCs is significantly increased, which stimulates the maturation of DCs and enhances the antitumor response [6]. Another clinical evaluation (NCT04613492) of recombinant NDV expressing IL-2 (MEDI9253) in combination with durvalumab in adult participants with select advanced/metastatic solid tumors is ongoing. Another strategy for further improving the NDV-modified vaccine was to combine it with dendritic cells. The Immune-Oncological Centre (IOZK) in Cologne (Germany) has received a permit for its NDV-modified dendritic cell vaccine IO-VACR in 2015. This is a specific, autologous, antitumor, directed DC vaccine for intracutaneous application. Overall, NDV has shown a variety of potential approaches in cancer therapy, including in its use alone, in combination with dendritic cell vaccines, in combination with standard treatments (e.g., temozolomide, TMZ), in combination with other immunotherapies, and in gene therapy. It is feasible to incorporate Newcastle disease virus (NDV) into combination immunotherapies and standard care, employing a multi-phase treatment strategy. This approach may have a good effect on malignant tumors that are prone to drug resistance and recurrence, such as GBM.

**Table 1:** Clinical trials of oncolytic NDV.

NDV Strains	Types of Cancer	Phase	Patients (*n*)	References
73-T	Melanoma	II	83	[123,124]
MTH-68/H	Advanced chemorefractory cancers	II	59	[125]
MTH-68/H	Glioblastoma multiforme (GBM), high-grade glioma	No data	4	[126]
PV701	Advanced solid cancers	I	113	[119,127]
HUJ	GBM	I/II	14	[128]
ATV-NDV	Early breast cancer, metastatic breast cancer, and metastatic ovarian cancer	I	121	[132]
ATV-NDV	Locally advanced renal cell carcinoma	II	208	[133]
ATV-NDV	Head and neck squamous cell carcinoma (HNSCC)	I	20	[134]
ATV-NDV	GBM	II	110	[135]
ATV-NDV	Colorectal resected carcinoma	II	57	[136]
ATV-NDV	Colorectal resected carcinoma	I	51	[137]
ATV-NDV	Colorectal cancer	III	310	[138]
ATV-NDV-αHN-αCD28	Colorectal cancer	I	40	[139]
MEDI5395 (rNDV-GM-CSF)	Advanced solid tumors	I	188	NCT03889275
MEDI9253 (rNDV-IL 12)	Solid tumors	I	86	NCT04613492

ATV-NDV: the NDV-modified autologous tumor vaccine; ATV-NDV-αHN-αCD28: the ATV-NDV strain expressing the anti-CD28 fusion protein, coupled to viral HN anchor molecules.

## 7. Conclusions

ND is a highly contagious poultry disease that has caused huge losses to the poultry industry in many countries. However, NDV, the causative agent of ND, offers many advantageous properties in terms of its development as a vaccine vector and in tumor treatment. Despite its potential, there are challenges in the broader application of NDV as a vaccine vector and oncolytic agent. These include the variability in NDV strains, the immune response to NDV in previously exposed animals and populations, and the delivery and stability of NDV-based therapeutics. Ongoing research is focused on optimizing NDV strains to improve oncolytic efficacy, enhance the immune-stimulatory properties of NDV-based vaccines, and develop effective delivery systems to target NDV to specific tissues or tumors. Its unique properties, coupled with advanced genetic engineering techniques, open up new avenues for the treatment and prevention of a wide range of diseases. Relevant basic research and clinical trials are being actively carried out, and it is believed that NDV will bring a new dawn for the treatment of infectious diseases and tumors in the near future.

## Data Availability

Not applicable.

## References

[1] Ganar K., Das M., Sinha S., Kumar S. (2014). Newcastle disease virus: Current status and our understanding. Virus Res..

[2] Burman B., Pesci G., Zamarin D. (2020). Newcastle Disease Virus at the Forefront of Cancer Immunotherapy. Cancers.

[3] Cuadrado-Castano S., Sanchez-Aparicio M.T., Garcia-Sastre A., Villar E. (2015). The therapeutic effect of death: Newcastle disease virus and its antitumor potential. Virus Res..

[4] Kan X., Yin Y., Song C., Tan L., Qiu X., Liao Y., Liu W., Meng S., Sun Y., Ding C. (2021). Newcastle disease virus induced ferroptosis through nutrient deprivation and ferritinophagy in tumor cells. iScience.

[5] Zhan Y., Yu S., Yang S., Qiu X., Meng C., Tan L., Song C., Liao Y., Liu W., Sun Y. (2020). Newcastle Disease virus infection activates PI3K/Akt/mTOR and p38 MAPK/Mnk1 pathways to benefit viral mRNA translation via interaction of the viral NP protein and host eIF4E. PLoS Pathog..

[6] Burke S., Shergold A., Elder M.J., Whitworth J., Cheng X., Jin H., Wilkinson R.W., Harper J., Carroll D.K. (2020). Oncolytic Newcastle disease virus activation of the innate immune response and priming of antitumor adaptive responses in vitro. Cancer Immunol. Immunother..

[7] Peeters B.P., de Leeuw O.S., Koch G., Gielkens A.L. (1999). Rescue of Newcastle disease virus from cloned cDNA: Evidence that cleavability of the fusion protein is a major determinant for virulence. J. Virol..

[8] Krishnamurthy S., Huang Z., Samal S.K. (2000). Recovery of a virulent strain of newcastle disease virus from cloned cDNA: Expression of a foreign gene results in growth retardation and attenuation. Virology.

[9] Jadhav A., Zhao L., Ledda A., Liu W., Ding C., Nair V.A.-O.X., Ferretti L. (2020). Patterns of RNA Editing in Newcastle Disease Virus Infections. Viruses.

[10] Huang Z., Krishnamurthy S., Panda A., Samal S.K. (2003). Newcastle disease virus V protein is associated with viral pathogenesis and functions as an alpha interferon antagonist. J. Virol..

[11] Park M.S., Garcia-Sastre A., Cros J.F., Basler C.F., Palese P. (2003). Newcastle disease virus V protein is a determinant of host range restriction. J. Virol..

[12] Nan F.L., Zhang H., Nan W.L., Xie C.Z., Ha Z., Chen X., Xu X.H., Qian J., Qiu X.S., Ge J.Y. (2021). Lentogenic NDV V protein inhibits IFN responses and represses cell apoptosis. Vet. Microbiol..

[13] Yang Y., Xue J., Teng Q., Li X., Bu Y., Zhang G. (2021). Mechanisms and consequences of Newcastle disease virus W protein subcellular localization in the nucleus or mitochondria. J. Virol..

[14] Tan L., Zhang Y., Zhan Y., Yuan Y., Sun Y., Qiu X., Meng C., Song C., Liao Y., Ding C. (2016). Newcastle disease virus employs macropinocytosis and Rab5a-dependent intracellular trafficking to infect DF-1 cells. Oncotarget.

[15] El-Sayed A., Harashima H. (2013). Endocytosis of gene delivery vectors: From clathrin-dependent to lipid raft-mediated endocytosis. Mol. Ther..

[16] Iorio R.M., Field G.M., Sauvron J.M., Mirza A.M., Deng R., Mahon P.J., Langedijk J.P. (2001). Structural and functional relationship between the receptor recognition and neuraminidase activities of the Newcastle disease virus hemagglutinin-neuraminidase protein: Receptor recognition is dependent on neuraminidase activity. J. Virol..

[17] Dimitrov K.M., Abolnik C., Afonso C.L., Albina E., Bahl J., Berg M., Briand F.X., Brown I.H., Choi K.S., Chvala I. (2019). Updated unified phylogenetic classification system and revised nomenclature for Newcastle disease virus. Infect. Genet. Evol..

[18] de Leeuw O.S., Koch G., Hartog L., Ravenshorst N., Peeters B.P.H. (2005). Virulence of Newcastle disease virus is determined by the cleavage site of the fusion protein and by both the stem region and globular head of the haemagglutinin-neuraminidase protein. J. Gen. Virol..

[19] Panda A., Huang Z., Elankumaran S., Rockemann D.D., Samal S.K. (2004). Role of fusion protein cleavage site in the virulence of Newcastle disease virus. Microb. Pathog..

[20] Wang Y., Yu W., Huo N., Wang W., Guo Y., Wei Q., Wang X., Zhang S., Yang Z., Xiao S.A.-O. (2017). Comprehensive analysis of amino acid sequence diversity at the F protein cleavage site of Newcastle disease virus in fusogenic activity. PLoS ONE.

[21] de Leeuw O.S., Hartog L., Koch G., Peeters B.P.H. (2003). Effect of fusion protein cleavage site mutations on virulence of Newcastle disease virus: Non-virulent cleavage site mutants revert to virulence after one passage in chicken brain. J. Gen. Virol..

[22] Schirrmacher V. (2016). Fifty Years of Clinical Application of Newcastle Disease Virus: Time to Celebrate!. Biomedicines.

[23] Cassel W.F., Garrett R.E. (1965). Newcastle disease virus as an antineoplastic agent. Cancer.

[24] Huang F., Dai C., Zhang Y., Zhao Y., Wang Y., Ru G. (2022). Development of Molecular Mechanisms and Their Application on Oncolytic Newcastle Disease Virus in Cancer Therapy. Front. Mol. Biosci..

[25] Al-Ziaydi A.G., Al-Shammari A.M., Hamzah M.I., Kadhim H.S., Jabir M.S. (2020). Hexokinase inhibition using D-Mannoheptulose enhances oncolytic newcastle disease virus-mediated killing of breast cancer cells. Cancer Cell Int..

[26] Al-Shammari A.M., Abdullah A.H., Allami Z.M., Yaseen N.Y. (2019). 2-Deoxyglucose and Newcastle Disease Virus Synergize to Kill Breast Cancer Cells by Inhibition of Glycolysis Pathway Through Glyceraldehyde3-Phosphate Downregulation. Front. Mol. Biosci..

[27] Reichard K.W., Lorence R.M., Cascino C.J., Peeples M.E., Walter R.J., Fernando M.B., Reyes H.M., Greager J.A. (1992). Newcastle disease virus selectively kills human tumor cells. J. Surg. Res..

[28] Garcia-Romero N., Palacin-Aliana I., Esteban-Rubio S., Madurga R., Rius-Rocabert S., Carrion-Navarro J., Presa J., Cuadrado-Castano S., Sanchez-Gomez P., Garcia-Sastre A. (2020). Newcastle Disease Virus (NDV) Oncolytic Activity in Human Glioma Tumors Is Dependent on CDKN2A-Type I IFN Gene Cluster Codeletion. Cells.

[29] Krishnamurthy S., Takimoto T., Scroggs R.A., Portner A. (2006). Differentially regulated interferon response determines the outcome of Newcastle disease virus infection in normal and tumor cell lines. J. Virol..

[30] Fiola C., Peeters B., Fournier P., Arnold A., Bucur M., Schirrmacher V. (2006). Tumor selective replication of Newcastle disease virus: Association with defects of tumor cells in antiviral defence. Int. J. Cancer.

[31] Schirrmacher V. (2022). Molecular Mechanisms of Anti-Neoplastic and Immune Stimulatory Properties of Oncolytic Newcastle Disease Virus. Biomedicines.

[32] Schirrmacher V., van Gool S., Stuecker W. (2019). Breaking Therapy Resistance: An Update on Oncolytic Newcastle Disease Virus for Improvements of Cancer Therapy. Biomedicines.

[33] Fournier P., Wilden H., Schirrmacher V. (2012). Importance of retinoic acid-inducible gene I and of receptor for type I interferon for cellular resistance to infection by Newcastle disease virus. Int. J. Oncol..

[34] Wilden H., Fournier P., Zawatzky R., Schirrmacher V. (2009). Expression of RIG-I, IRF3, IFN-beta and IRF7 determines resistance or susceptibility of cells to infection by Newcastle Disease Virus. Int. J. Oncol..

[35] Washburn B., Schirrmacher V. (2002). Human tumor cell infection by Newcastle Disease Virus leads to upregulation of HLA and cell adhesion molecules and to induction of interferons, chemokines and finally apoptosis. Int. J. Oncol..

[36] Lenza M.P., Atxabal U., Oyenarte I., Jiménez-Barbero J., Ereño-Orbea J. (2020). Current Status on Therapeutic Molecules Targeting Siglec Receptors. Cells.

[37] Mansour M., Palese P., Zamarin D. (2011). Oncolytic specificity of Newcastle disease virus is mediated by selectivity for apoptosis-resistant cells. J. Virol..

[38] Lazar I., Yaacov B., Shiloach T., Eliahoo E., Kadouri L., Lotem M., Perlman R., Zakay-Rones Z., Panet A., Ben-Yehuda D. (2010). The oncolytic activity of Newcastle disease virus NDV-HUJ on chemoresistant primary melanoma cells is dependent on the proapoptotic activity of the inhibitor of apoptosis protein Livin. J. Virol..

[39] Puhlmann J., Puehler F., Mumberg D., Boukamp P., Beier R. (2010). Rac1 is required for oncolytic NDV replication in human cancer cells and establishes a link between tumorigenesis and sensitivity to oncolytic virus. Oncogene.

[40] Mustafa Z., Shamsuddin H.S., Ideris A., Ibrahim R., Jaafar H., Ali A.M., Abdullah J.M. (2013). Viability reduction and Rac1 gene downregulation of heterogeneous ex-vivo glioma acute slice infected by the oncolytic Newcastle disease virus strain V4UPM. Biomed. Res. Int..

[41] Kent L.N., Leone G. (2019). The broken cycle: E2F dysfunction in cancer. Nat. Rev. Cancer.

[42] D’Arcy M.S. (2019). Cell death: A review of the major forms of apoptosis, necrosis and autophagy. Cell Biol. Int..

[43] Shalini S., Dorstyn L., Dawar S., Kumar S. (2015). Old, new and emerging functions of caspases. Cell Death Differ..

[44] Kaloni D., Diepstraten S.T., Strasser A., Kelly G.L. (2023). BCL-2 protein family: Attractive targets for cancer therapy. Apoptosis.

[45] Ravindra P.V., Tiwari A.K., Ratta B., Bais M.V., Chaturvedi U., Palia S.K., Sharma B., Chauhan R.S. (2009). Time course of Newcastle disease virus-induced apoptotic pathways. Virus Res..

[46] Molouki A., Hsu Y.T., Jahanshiri F., Abdullah S., Rosli R., Yusoff K. (2011). The matrix (M) protein of Newcastle disease virus binds to human bax through its BH3 domain. Virol. J..

[47] Cuadrado-Castano S., Ayllon J., Mansour M., de la Iglesia-Vicente J., Jordan S., Tripathi S., García-Sastre A., Villar E. (2015). Enhancement of the proapoptotic properties of newcastle disease virus promotes tumor remission in syngeneic murine cancer models. Mol. Cancer Ther..

[48] Bai F.L., Yu Y.H., Tian H., Ren G.P., Wang H., Zhou B., Han X.H., Yu Q.Z., Li D.S. (2014). Genetically engineered Newcastle disease virus expressing interleukin-2 and TNF-related apoptosis-inducing ligand for cancer therapy. Cancer Biol. Ther..

[49] Kazimirsky G., Jiang W., Slavin S., Ziv-Av A., Brodie C. (2016). Mesenchymal stem cells enhance the oncolytic effect of Newcastle disease virus in glioma cells and glioma stem cells via the secretion of TRAIL. Stem Cell Res. Ther..

[50] Ch’ng W.C., Abd-Aziz N., Ong M.H., Stanbridge E.J., Shafee N. (2015). Human renal carcinoma cells respond to Newcastle disease virus infection through activation of the p38 MAPK/NF-κB/IκBα pathway. Cell. Oncol..

[51] Li Y., Jiang W., Niu Q., Sun Y., Meng C., Tan L., Song C., Qiu X., Liao Y., Ding C. (2019). eIF2α-CHOP-BCl-2/JNK and IRE1α-XBP1/JNK signaling promote apoptosis and inflammation and support the proliferation of Newcastle disease virus. Cell Death Dis..

[52] Elankumaran S., Rockemann D., Samal S.K. (2006). Newcastle disease virus exerts oncolysis by both intrinsic and extrinsic caspase-dependent pathways of cell death. J. Virol..

[53] Zeng J., Fournier P., Schirrmacher V. (2002). Induction of interferon-alpha and tumor necrosis factor-related apoptosis-inducing ligand in human blood mononuclear cells by hemagglutinin-neuraminidase but not F protein of Newcastle disease virus. Virology.

[54] Liao Y., Wang H.X., Mao X., Fang H., Wang H., Li Y., Sun Y., Meng C., Tan L., Song C. (2017). RIP1 is a central signaling protein in regulation of TNF-α/TRAIL mediated apoptosis and necroptosis during Newcastle disease virus infection. Oncotarget.

[55] Ghrici M., El Zowalaty M., Omar A.R., Ideris A. (2013). Newcastle disease virus Malaysian strain AF2240 induces apoptosis in MCF-7 human breast carcinoma cells at an early stage of the virus life cycle. Int. J. Mol. Med..

[56] He D., Sun L., Li C., Hu N., Sheng Y., Chen Z., Li X., Chi B., Jin N. (2014). Anti-tumor effects of an oncolytic adenovirus expressing hemagglutinin-neuraminidase of Newcastle disease virus in vitro and in vivo. Viruses.

[57] Molouki A., Yusoff K. (2012). NDV-induced apoptosis in absence of Bax; evidence of involvement of apoptotic proteins upstream of mitochondria. Virol. J..

[58] Shan P., Tang B., Xie S., Zhang Z., Fan J., Wei Z., Song C. (2021). NDV-D90 inhibits 17β-estradiol-mediated resistance to apoptosis by differentially modulating classic and nonclassic estrogen receptors in breast cancer cells. J. Cell Biochem..

[59] Fan X., Lu H., Cui Y., Hou X., Huang C., Liu G. (2018). Overexpression of p53 delivered using recombinant NDV induces apoptosis in glioma cells by regulating the apoptotic signaling pathway. Exp. Ther. Med..

[60] Li X., Jia Y., Liu H., Wang X., Chu Z., Hu R., Ren J., Xiao S., Zhang S., Wang X. (2019). High level expression of ISG12(1) promotes cell apoptosis via mitochondrial-dependent pathway and so as to hinder Newcastle disease virus replication. Vet. Microbiol..

[61] Wang C., Chu Z., Liu W., Pang Y., Gao X., Tang Q., Ma J., Lu K., Adam F.E.A., Dang R. (2018). Newcastle disease virus V protein inhibits apoptosis in DF-1 cells by downregulating TXNL1. Vet. Res..

[62] Peker N., Gozuacik D. (2020). Autophagy as a Cellular Stress Response Mechanism in the Nervous System. J. Mol. Biol..

[63] Russell R.C., Yuan H.X., Guan K.L. (2014). Autophagy regulation by nutrient signaling. Cell Res..

[64] Meng C., Zhou Z., Jiang K., Yu S., Jia L., Wu Y., Liu Y., Meng S., Ding C. (2012). Newcastle disease virus triggers autophagy in U251 glioma cells to enhance virus replication. Arch. Virol..

[65] Koks C.A., Garg A.D., Ehrhardt M., Riva M., Vandenberk L., Boon L., De Vleeschouwer S., Agostinis P., Graf N., Van Gool S.W. (2015). Newcastle disease virotherapy induces long-term survival and tumor-specific immune memory in orthotopic glioma through the induction of immunogenic cell death. Int. J. Cancer.

[66] Sun Y., Yu S., Ding N., Meng C., Meng S., Zhang S., Zhan Y., Qiu X., Tan L., Chen H. (2014). Autophagy benefits the replication of Newcastle disease virus in chicken cells and tissues. J. Virol..

[67] Cheng J.H., Sun Y.J., Zhang F.Q., Zhang X.R., Qiu X.S., Yu L.P., Wu Y.T., Ding C. (2016). Newcastle disease virus NP and P proteins induce autophagy via the endoplasmic reticulum stress-related unfolded protein response. Sci. Rep..

[68] Ren S., Rehman Z.U., Shi M., Yang B., Qu Y., Yang X.F., Shao Q., Meng C., Yang Z., Gao X. (2019). Syncytia generated by hemagglutinin-neuraminidase and fusion proteins of virulent Newcastle disease virus induce complete autophagy by activating AMPK-mTORC1-ULK1 signaling. Vet. Microbiol..

[69] Bu X., Zhao Y., Zhang Z., Wang M., Li M., Yan Y. (2016). Recombinant Newcastle disease virus (rL-RVG) triggers autophagy and apoptosis in gastric carcinoma cells by inducing ER stress. Am. J. Cancer Res..

[70] Yan Y., Shao X., Gu W., Zhang A., Bu X., Liang B. (2020). Recombinant virus expressing hIFN-λ1 (rL-hIFN-λ1) has important effects on endoplasmic reticulum stress, autophagy and apoptosis in small cell lung cancer. Transl. Cancer Res..

[71] Chen X., Cubillos-Ruiz J.R. (2021). Endoplasmic reticulum stress signals in the tumour and its microenvironment. Nat. Rev. Cancer.

[72] Jiang P., Mizushima N. (2015). LC3- and p62-based biochemical methods for the analysis of autophagy progression in mammalian cells. Methods.

[73] Gong Y., Tang N., Liu P., Sun Y., Lu S., Liu W., Tan L., Song C., Qiu X., Liao Y. (2022). Newcastle disease virus degrades SIRT3 via PINK1-PRKN-dependent mitophagy to reprogram energy metabolism in infected cells. Autophagy.

[74] Meng S., Zhou Z., Chen F., Kong X., Liu H., Jiang K., Liu W., Hu M., Zhang X., Ding C. (2012). Newcastle disease virus induces apoptosis in cisplatin-resistant human lung adenocarcinoma A549 cells in vitro and in vivo. Cancer Lett..

[75] Kang Y., Yuan R., Xiang B., Zhao X., Gao P., Dai X., Liao M., Ren T. (2017). Newcastle disease virus-induced autophagy mediates antiapoptotic signaling responses in vitro and in vivo. Oncotarget.

[76] Xu H.D., Qin Z.H. (2019). Beclin 1, Bcl-2 and Autophagy. Adv. Exp. Med. Biol..

[77] Jarahian M., Watzl C., Fournier P., Arnold A., Djandji D., Zahedi S., Cerwenka A., Paschen A., Schirrmacher V., Momburg F. (2009). Activation of natural killer cells by newcastle disease virus hemagglutinin-neuraminidase. J. Virol..

[78] Washburn B., Weigand M.A., Grosse-Wilde A., Janke M., Stahl H., Rieser E., Sprick M.R., Schirrmacher V., Walczak H. (2003). TNF-related apoptosis-inducing ligand mediates tumoricidal activity of human monocytes stimulated by Newcastle disease virus. J. Immunol..

[79] Schirrmacher V., Bai L., Umansky V., Yu L., Xing Y., Qian Z. (2000). Newcastle disease virus activates macrophages for anti-tumor activity. Int. J. Oncol..

[80] Zhao L., Niu C., Shi X., Xu D., Li M., Cui J., Li W., Xu J., Jin H. (2018). Dendritic cells loaded with the lysate of tumor cells infected with Newcastle Disease Virus trigger potent anti-tumor immunity by promoting the secretion of IFN-γ and IL-2 from T cells. Oncol. Lett..

[81] Elankumaran S., Chavan V., Qiao D., Shobana R., Moorkanat G., Biswas M., Samal S.K. (2010). Type I interferon-sensitive recombinant newcastle disease virus for oncolytic virotherapy. J. Virol..

[82] Shortman K., Heath W.R. (2010). The CD8^+^ dendritic cell subset. Immunol. Rev..

[83] Fuertes M.B., Kacha A.K., Kline J., Woo S.R., Kranz D.M., Murphy K.M., Gajewski T.F. (2011). Host type I IFN signals are required for antitumor CD8^+^ T cell responses through CD8{alpha}^+^ dendritic cells. J. Exp. Med..

[84] Buijs P.R., van Eijck C.H., Hofland L.J., Fouchier R.A., van den Hoogen B.G. (2014). Different responses of human pancreatic adenocarcinoma cell lines to oncolytic Newcastle disease virus infection. Cancer Gene Ther..

[85] Hu Z., Ni J., Cao Y., Liu X. (2020). Newcastle Disease Virus as a Vaccine Vector for 20 Years: A Focus on Maternally Derived Antibody Interference. Vaccines.

[86] Kim S.H., Samal S.K. (2019). Innovation in Newcastle Disease Virus Vectored Avian Influenza Vaccines. Viruses.

[87] Fulber J.P.C., Kamen A.A. (2022). Development and Scalable Production of Newcastle Disease Virus-Vectored Vaccines for Human and Veterinary Use. Viruses.

[88] Bello M.B., Yusoff K., Ideris A., Hair-Bejo M., Jibril A.H., Peeters B.P.H., Omar A.R. (2020). Exploring the Prospects of Engineered Newcastle Disease Virus in Modern Vaccinology. Viruses.

[89] Saikia D.P., Yadav K., Pathak D.C., Ramamurthy N., D’Silva A.L., Marriappan A.K., Ramakrishnan S., Vakharia V.N., Chellappa M.M., Dey S. (2019). Recombinant Newcastle Disease Virus (NDV) Expressing Sigma C Protein of Avian Reovirus (ARV) Protects against Both ARV and NDV in Chickens. Pathogens.

[90] Abozeid H.H., Paldurai A., Varghese B.P., Khattar S.K., Afifi M.A., Zouelfakkar S., El-Deeb A.H., El-Kady M.F., Samal S.K. (2019). Development of a recombinant Newcastle disease virus-vectored vaccine for infectious bronchitis virus variant strains circulating in Egypt. Vet. Res..

[91] Shirvani E., Paldurai A., Manoharan V.K., Varghese B.P., Samal S.K. (2018). A Recombinant Newcastle Disease Virus (NDV) Expressing S Protein of Infectious Bronchitis Virus (IBV) Protects Chickens against IBV and NDV. Sci. Rep..

[92] Zeng Z., He Y., Wang Z., Yao L., Li L., Shang Y., Wang H., Zhang R., Shao H., Luo Q. (2023). Characterization of a Recombinant Thermostable Newcastle Disease Virus (NDV) Expressing Glycoprotein gB of Infectious Laryngotracheitis Virus (ILTV) Protects Chickens against ILTV Challenge. Viruses.

[93] Qiao Q., Song M., Song C., Zhang Y., Wang X., Huang Q., Wang B., Yang P., Zhao S., Li Y. (2021). Single-Dose Vaccination of Recombinant Chimeric Newcastle Disease Virus (NDV) LaSota Vaccine Strain Expressing Infectious Bursal Disease Virus (IBDV) VP2 Gene Provides Full Protection against Genotype VII NDV and IBDV Challenge. Vaccines.

[94] Sun W.Y., Cao X.L., Wang Y.X., Guo X.C., Liu J.M., Xue Z.Q., Li H.J., Wang W., Zhang T.T., Li Q. (2024). Development and evaluation of a bivalent vaccine based on recombinant newcastle disease virus expressing infectious bursal disease virus VP2L-CH3-CH4 in SPF chickens. Vet. Microbiol..

[95] Tian K.Y., Guo H.F., Li N., Zhang Y.H., Wang Z., Wang B., Yang X., Li Y.T., Zhao J. (2020). Protection of chickens against hepatitis-hydropericardium syndrome and Newcastle disease with a recombinant Newcastle disease virus vaccine expressing the fowl adenovirus serotype 4 fiber-2 protein. Vaccine.

[96] Sun M., Dong J., Li L., Lin Q., Sun J., Liu Z., Shen H., Zhang J., Ren T., Zhang C. (2018). Recombinant Newcastle disease virus (NDV) expressing Duck Tembusu virus (DTMUV) pre-membrane and envelope proteins protects ducks against DTMUV and NDV challenge. Vet. Microbiol..

[97] Wang J., Cong Y., Yin R., Feng N., Yang S., Xia X., Xiao Y., Wang W., Liu X., Hu S. (2015). Generation and evaluation of a recombinant genotype VII Newcastle disease virus expressing VP3 protein of Goose parvovirus as a bivalent vaccine in goslings. Virus Res..

[98] Xu D., Li C., Liu G., Chen Z., Jia R. (2019). Generation and evaluation of a recombinant goose origin Newcastle disease virus expressing Cap protein of goose origin avastrovirus as a bivalent vaccine in goslings. Poult. Sci..

[99] Kumar R., Kumar V., Kekungu P., Barman N.N., Kumar S. (2019). Evaluation of surface glycoproteins of classical swine fever virus as immunogens and reagents for serological diagnosis of infections in pigs: A recombinant Newcastle disease virus approach. Arch. Virol..

[100] Zhang H., Nan F., Li Z., Zhao G., Xie C., Ha Z., Zhang J., Han J., Xiao P., Zhuang X. (2019). Construction and immunological evaluation of recombinant Newcastle disease virus vaccines expressing highly pathogenic porcine reproductive and respiratory syndrome virus GP3/GP5 proteins in pigs. Vet. Microbiol..

[101] Zhang M., Ge J., Wen Z., Chen W., Wang X., Liu R., Bu Z. (2017). Characterization of a recombinant Newcastle disease virus expressing the glycoprotein of bovine ephemeral fever virus. Arch. Virol..

[102] Ge J., Wang X., Tian M., Gao Y., Wen Z., Yu G., Zhou W., Zu S., Bu Z. (2015). Recombinant Newcastle disease viral vector expressing hemagglutinin or fusion of canine distemper virus is safe and immunogenic in minks. Vaccine.

[103] Ge J., Wang X., Tao L., Wen Z., Feng N., Yang S., Xia X., Yang C., Chen H., Bu Z. (2011). Newcastle disease virus-vectored rabies vaccine is safe, highly immunogenic, and provides long-lasting protection in dogs and cats. J. Virol..

[104] Tcheou J., Raskin A., Singh G., Kawabata H., Bielak D., Sun W., González-Domínguez I., Sather D.N., García-Sastre A., Palese P. (2021). Safety and Immunogenicity Analysis of a Newcastle Disease Virus (NDV-HXP-S) Expressing the Spike Protein of SARS-CoV-2 in Sprague Dawley Rats. Front. Immunol..

[105] Jung B.K., An Y.H., Jang J.J., Jeon J.H., Jang S.H., Jang H. (2022). The human ACE-2 receptor binding domain of SARS-CoV-2 express on the viral surface of the Newcastle disease virus as a non-replicating viral vector vaccine candidate. PLoS ONE.

[106] Zhao W., Zhang P., Bai S., Lv M., Wang J., Chen W., Yu Q., Wu J. (2021). Heterologous prime-boost regimens with HAdV-5 and NDV vectors elicit stronger immune responses to Ebola virus than homologous regimens in mice. Arch. Virol..

[107] Khattar S.K., DeVico A.L., LaBranche C.C., Panda A., Montefiori D.C., Samal S.K. (2016). Enhanced Immune Responses to HIV-1 Envelope Elicited by a Vaccine Regimen Consisting of Priming with Newcastle Disease Virus Expressing HIV gp160 and Boosting with gp120 and SOSIP gp140 Proteins. J. Virol..

[108] Nath B., Vandna, Saini H.M., Prasad M., Kumar S. (2020). Evaluation of Japanese encephalitis virus E and NS1 proteins immunogenicity using a recombinant Newcastle disease virus in mice. Vaccine.

[109] Viktorova E.G., Khattar S.K., Kouiavskaia D., Laassri M., Zagorodnyaya T., Dragunsky E., Samal S., Chumakov K., Belov G.A. (2018). Newcastle Disease Virus-Based Vectored Vaccine against Poliomyelitis. J. Virol..

[110] Song K.Y., Wong J., Gonzalez L., Sheng G., Zamarin D., Fong Y. (2010). Antitumor efficacy of viral therapy using genetically engineered Newcastle disease virus [NDV(F3aa)-GFP] for peritoneally disseminated gastric cancer. J. Mol. Med..

[111] Bu X., Li M., Zhao Y., Liu S., Wang M., Ge J., Bu Z., Yan Y. (2016). Genetically engineered Newcastle disease virus expressing human interferon-λ1 induces apoptosis in gastric adenocarcinoma cells and modulates the Th1/Th2 immune response. Oncol. Rep..

[112] Yan Y., Liang B., Zhang J., Liu Y., Bu X. (2015). Apoptotic induction of lung adenocarcinoma *A*549 cells infected by recombinant RVG Newcastle disease virus (rL-RVG) in vitro. Mol. Med. Rep..

[113] Hu L., Sun S., Wang T., Li Y., Jiang K., Lin G., Ma Y., Barr M.P., Song F., Zhang G. (2015). Oncolytic newcastle disease virus triggers cell death of lung cancer spheroids and is enhanced by pharmacological inhibition of autophagy. Am. J. Cancer Res..

[114] Keshavarz M., Nejad A.S.M., Esghaei M., Bokharaei-Salim F., Dianat-Moghadam H., Keyvani H., Ghaemi A. (2020). Oncolytic Newcastle disease virus reduces growth of cervical cancer cell by inducing apoptosis. Saudi J. Biol. Sci..

[115] Wang X., Shao X., Gu L., Jiang K., Wang S., Chen J., Fang J., Guo X., Yuan M., Shi J. (2020). Targeting STAT3 enhances NDV-induced immunogenic cell death in prostate cancer cells. J. Cell Mol. Med..

[116] Song H., Zhong L.P., He J., Huang Y., Zhao Y.X. (2019). Application of Newcastle disease virus in the treatment of colorectal cancer. World J. Clin. Cases.

[117] Abdullah J.M., Mustafa Z., Ideris A. (2014). Newcastle disease virus interaction in targeted therapy against proliferation and invasion pathways of glioblastoma multiforme. Biomed. Res. Int..

[118] Wu Y.-Y., Sun T.-K., Chen M.-S., Munir M., Liu H.-J. (2023). Oncolytic viruses-modulated immunogenic cell death, apoptosis and autophagy linking to virotherapy and cancer immune response. Front. Cell. Infect. Microbiol..

[119] Csatary L.K., Moss R.W., Beuth J., Töröcsik B., Szeberenyi J., Bakacs T. (1999). Beneficial treatment of patients with advanced cancer using a Newcastle disease virus vaccine (MTH-68/H). Anticancer Res..

[120] Pecora A.L., Rizvi N., Cohen G.I., Meropol N.J., Sterman D., Marshall J.L., Goldberg S., Gross P., O’Neil J.D., Groene W.S. (2002). Phase I trial of intravenous administration of PV701, an oncolytic virus, in patients with advanced solid cancers. J. Clin. Oncol..

[121] Yaacov B., Eliahoo E., Lazar I., Ben-Shlomo M., Greenbaum I., Panet A., Zakay-Rones Z. (2008). Selective oncolytic effect of an attenuated Newcastle disease virus (NDV-HUJ) in lung tumors. Cancer Gene Ther..

[122] Schirrmacher V., Fournier P. (2009). Newcastle disease virus: A promising vector for viral therapy, immune therapy, and gene therapy of cancer. Methods Mol. Biol..

[123] Wheelock E.F., Dingle J.H. (1964). Observations on the repeated administration of viruses to a patient with acute leukemia. N. Engl. J. Med..

[124] Cassel W.A., Murray D.R. (1992). A ten-year follow-up on stage II malignant melanoma patients treated postsurgically with Newcastle disease virus oncolysate. Med. Oncol. Tumor Pharmacother..

[125] Batliwalla F.M., Bateman B.A., Serrano D., Murray D., Macphail S., Maino V.C., Ansel J.C., Gregersen P.K., Armstrong C.A. (1998). A 15-year follow-up of AJCC stage III malignant melanoma patients treated postsurgically with Newcastle disease virus (NDV) oncolysate and determination of alterations in the CD8 T cell repertoire. Mol. Med..

[126] Csatary L.K., Eckhardt S., Bukosza I., Czegledi F., Fenyvesi C., Gergely P., Bodey B., Csatary C.M. (1993). Attenuated veterinary virus vaccine for the treatment of cancer. Cancer Detect. Prev..

[127] Csatary L.K., Gosztonyi G., Szeberenyi J., Fabian Z., Liszka V., Bodey B., Csatary C.M. (2004). MTH-68/H oncolytic viral treatment in human high-grade gliomas. J. Neurooncol.

[128] Lorence R.M., Pecora A.L., Major P.P., Hotte S.J., Laurie S.A., Roberts M.S., Groene W.S., Bamat M.K. (2003). Overview of phase I studies of intravenous administration of PV701, an oncolytic virus. Curr. Opin. Mol. Ther..

[129] Freeman A.I., Zakay-Rones Z., Gomori J.M., Linetsky E., Rasooly L., Greenbaum E., Rozenman-Yair S., Panet A., Libson E., Irving C.S. (2006). Phase I/II trial of intravenous NDV-HUJ oncolytic virus in recurrent glioblastoma multiforme. Mol. Ther..

[130] Kalyanasundram J., Hamid A., Yusoff K., Chia S.L. (2018). Newcastle disease virus strain AF2240 as an oncolytic virus: A review. Acta Trop..

[131] Najmuddin S., Amin Z.M., Tan S.W., Yeap S.K., Kalyanasundram J., Ani M.A.C., Veerakumarasivam A., Chan S.C., Chia S.L., Yusoff K. (2020). Cytotoxicity study of the interleukin-12-expressing recombinant Newcastle disease virus strain, rAF-IL12, towards CT26 colon cancer cells in vitro and in vivo. Cancer Cell Int..

[132] Syed Najmuddin S.U.F., Amin Z.M., Tan S.W., Yeap S.K., Kalyanasundram J., Veerakumarasivam A., Chan S.C., Chia S.L., Yusoff K., Alitheen N.B. (2020). Oncolytic effects of the recombinant Newcastle disease virus, rAF-IL12, against colon cancer cells in vitro and in tumor-challenged NCr-Foxn1nu nude mice. PeerJ.

[133] Ahlert T., Sauerbrei W., Bastert G., Ruhland S., Bartik B., Simiantonaki N., Schumacher J., Häcker B., Schumacher M., Schirrmacher V. (1997). Tumor-cell number and viability as quality and efficacy parameters of autologous virus-modified cancer vaccines in patients with breast or ovarian cancer. J. Clin. Oncol..

[134] Kirchner H.H., Anton P., Atzpodien J. (1995). Adjuvant treatment of locally advanced renal cancer with autologous virus-modified tumor vaccines. World J. Urol..

[135] Karcher J., Dyckhoff G., Beckhove P., Reisser C., Brysch M., Ziouta Y., Helmke B.H., Weidauer H., Schirrmacher V., Herold-Mende C. (2004). Antitumor vaccination in patients with head and neck squamous cell carcinomas with autologous virus-modified tumor cells. Cancer Res..

[136] Steiner H.H., Bonsanto M.M., Beckhove P., Brysch M., Geletneky K., Ahmadi R., Schuele-Freyer R., Kremer P., Ranaie G., Matejic D. (2004). Antitumor vaccination of patients with glioblastoma multiforme: A pilot study to assess feasibility, safety, and clinical benefit. J. Clin. Oncol..

[137] Ockert D., Schirrmacher V., Beck N., Stoelben E., Ahlert T., Flechtenmacher J., Hagmüller E., Buchcik R., Nagel M., Saeger H.D. (1996). Newcastle disease virus-infected intact autologous tumor cell vaccine for adjuvant active specific immunotherapy of resected colorectal carcinoma. Clin. Cancer Res..

[138] Schulze T., Kemmner W., Weitz J., Wernecke K.D., Schirrmacher V., Schlag P.M. (2009). Efficiency of adjuvant active specific immunization with Newcastle disease virus modified tumor cells in colorectal cancer patients following resection of liver metastases: Results of a prospective randomized trial. Cancer Immunol. Immunother..

[139] Liang W., Wang H., Sun T.M., Yao W.Q., Chen L.L., Jin Y., Li C.L., Meng F.J. (2003). Application of autologous tumor cell vaccine and NDV vaccine in treatment of tumors of digestive tract. World J. Gastroenterol..

[140] Schirrmacher V., Schlude C., Weitz J., Beckhove P. (2015). Strong T-cell costimulation can reactivate tumor antigen-specific T cells in late-stage metastasized colorectal carcinoma patients: Results from a phase I clinical study. Int. J. Oncol..

[141] Numpadit S., Ito C., Nakaya T., Hagiwara K. (2023). Investigation of oncolytic effect of recombinant Newcastle disease virus in primary and metastatic oral melanoma. Med. Oncol..

[142] Ren G., Tian G., Liu Y., He J., Gao X., Yu Y., Liu X., Zhang X., Sun T., Liu S. (2016). Recombinant Newcastle Disease Virus Encoding IL-12 and/or IL-2 as Potential Candidate for Hepatoma Carcinoma Therapy. Technol. Cancer Res. Treat..

[143] Xu X., Sun Q., Mei Y., Liu Y., Zhao L. (2018). Newcastle disease virus co-expressing interleukin 7 and interleukin 15 modified tumor cells as a vaccine for cancer immunotherapy. Cancer Sci..

[144] Xu X., Yi C., Yang X., Xu J., Sun Q., Liu Y., Zhao L. (2019). Tumor Cells Modified with Newcastle Disease Virus Expressing IL-24 as a Cancer Vaccine. Mol. Ther. Oncolytics.

[145] Huang F.Y., Wang J.Y., Dai S.Z., Lin Y.Y., Sun Y., Zhang L., Lu Z., Cao R., Tan G.H. (2020). A recombinant oncolytic Newcastle virus expressing MIP-3α promotes systemic antitumor immunity. J. Immunother. Cancer.

[146] Tian L., Liu T., Jiang S., Cao Y., Kang K., Su H., Ren G., Wang Z., Xiao W., Li D. (2023). Oncolytic Newcastle disease virus expressing the co-stimulator OX40L as immunopotentiator for colorectal cancer therapy. Gene Ther..

[147] Nettelbeck D.M., Leber M.F., Altomonte J., Angelova A., Beil J., Berchtold S., Delic M., Eberle J., Ehrhardt A., Engeland C.E. (2021). Virotherapy in Germany-Recent Activities in Virus Engineering, Preclinical Development, and Clinical Studies. Viruses.

[148] Vijayakumar G., Palese P., Goff P.H. (2019). Oncolytic Newcastle disease virus expressing a checkpoint inhibitor as a radioenhancing agent for murine melanoma. EBioMedicine.

[149] Stewart R., Morrow M., Hammond S.A., Mulgrew K., Marcus D., Poon E., Watkins A., Mullins S., Chodorge M., Andrews J. (2015). Identification and Characterization of MEDI4736, an Antagonistic Anti-PD-L1 Monoclonal Antibody. Cancer Immunol. Res..

